# Regulation of Pancreatic microRNA-7 Expression

**DOI:** 10.1155/2012/695214

**Published:** 2012-05-17

**Authors:** Sharon Kredo-Russo, Avital Ness, Amitai D. Mandelbaum, Michael D. Walker, Eran Hornstein

**Affiliations:** ^1^Department of Molecular Genetics, Weizmann Institute of Science, Rehovot 76100, Israel; ^2^Department of Biological Chemistry, Weizmann Institute of Science, Rehovot 76100, Israel

## Abstract

Genome-encoded microRNAs (miRNAs) provide a posttranscriptional regulatory layer, which is important for pancreas development. Differentiation of endocrine cells is controlled by a network of pancreatic transcription factors including Ngn3 and NeuroD/Beta2. However, how specific miRNAs are intertwined into this transcriptional network is not well understood. Here, we characterize the regulation of microRNA-7 (miR-7) by endocrine-specific transcription factors. Our data reveal that three independent miR-7 genes are coexpressed in the pancreas. We have identified conserved blocks upstream of pre-miR-7a-2 and pre-miR-7b and demonstrated by functional assays that they possess promoter activity, which is increased by the expression of NeuroD/Beta2. These data suggest that the endocrine specificity of miR-7 expression is governed by transcriptional mechanisms and involves members of the pancreatic endocrine network of transcription factors.

## 1. Introduction

The development of the endocrine pancreas is governed by a network of transcription factors that specify different endocrine cell types, including insulin-producing beta cells, glucagon-producing alpha cells, somatostatin-producing delta cells, pancreatic polypeptide-producing PP cells, and ghrelin-producing epsilon cells [1–3]. The endocrine differentiation program is initiated by neurogenin3 (Ngn3) [4, 5]. Next, a complex network of transcription factors is activated to differentially specify the endocrine lineages (reviewed in [3, 6]).

Genome-encoded miRNAs act in concert with transcription factors to refine gene expression and confer robustness to developmental transitions [7–10]. Many miRNA genes are nested within introns of protein-coding genes and are subjected to transcriptional control with their host gene [11]. However, other miRNA genes are located in intergenic regions and are expressed autonomously. For example, in a previous study, we characterized the pancreas-enriched miR-375 and demonstrated that cell specificity is controlled transcriptionally through well-defined cis-regulatory elements [12].

Like miR-375, miR-7 is highly and selectively expressed in the endocrine pancreas of zebrafish, mouse, and human [13–16]. miR-7 is an evolutionarily conserved miRNA, encoded by a single gene in flies and by three different genomic loci in mammals. In mice two miR-7 genes are located in intergenic regions of Chr. 7 (mmu-mir-7a-2) and Chr. 17 (mmu-mir-7b), whereas a third miR-7 gene, mmu-mir-7a-1, is embedded within an intron of the gene encoding for the RNA-binding protein, Hnrnpk (MGI: 99894, on Chr. 13).

The two miR-7a genes generate an identical 22nt mature sequence, whereas miR-7b differs by a single nucleotide. However, functionally the three genes are identical, as they harbor the same “seed” sequence. Hence, all miR-7 genes coregulate the very same target set. While a defined set of targets is suggested for miR-7 [17] some of which have been experimentally validated [18], little is known about miR-7 promoter structure or the mechanisms controlling miR-7 expression.

In this study we characterized elements within the mmu-mir-7a-2 and mmu-mir-7b upstream regulatory sequences. We show that miR-7 responds to Ngn3 directly. However, our data suggest that NeuroD/Beta2, the primary effector of Ngn3, controls miR-7 and is probably responsible for maintenance of miR-7 expression in differentiated endocrine cells.

## 2. Materials and Methods

### 2.1. Quantitative PCR for Precursor and Mature miRNA

Extraction of total RNA was carried out by the miRNeasy Mini Kit (Qiagen). For precursor quantification, synthesis of cDNA was performed using miScript system (Qiagen). cDNA was synthesized from miRNAs using Taqman MicroRNA qPCR Assays (Applied Biosystems). qPCR analysis of mRNA was performed on LightCycler 480 System (Roche) using Kapa SYBR Green qPCR kit (Finnzymes). miRNA levels were normalized to the expression of small RNAs (sno234 and U6) and mRNA normalized to GAPDH and HPRT. (primer sequences are described in Supplementary Table 1 available online at doi: 10.1155/2012/695214).

### 2.2. Cell Culture and Luciferase Reporter Assay

HEK-293T cells (American Type Culture Collection), betaTC-3 (a gift from Shimon Efrat), and MIN6 cells (a gift from Jun-ichi Miyazaki) were grown in Dulbecco's Modified Eagle Medium (DMEM) with 10% FBS, 2 mM L-glutamine, 100 U/mL penicillin/streptomycin at 37°C at 5% CO_2_ in a humidified incubator. Experiments on MIN6 cells were performed between passages 18 to 28.

For miR-7 promoter analyses, fragments of 256 bp, 206 bp, and 156 bp (representing miR-7a-2 “block 1”, miR-7a-2 “block 2”, and “block 3” of miR-7b, resp.) were subcloned into pGL3-basic, using restriction enzymes BglII and KpnI. Primer sequences are described in Table  S1. HEK-293T cells were transfected with 100 ng of the reporter, 20 ng of A20 Renilla reporter, and in addition 50 ng of pcDNA3 empty and/or NeuroD/Beta2 and Ngn3 expression vectors. Reporter activity was measured 48 h after transfection with the Dual-Luciferase Reporter Assay System (Promega).

For overexpression analysis, expression vectors for transcription factors, pcDNA3 empty vector, and CMV-GFP vectors were transfected to MIN6 cells using Lipofectamine 2000 reagent (Invitrogen), according to the manufacture's instructions. miR-7 endogenous expression was analyzed by qPCR 48 h later.

### 2.3. Statistical Analysis

Analysis was performed using either Student's  *t-*test or two-way ANOVA by the JMP software. Results are given as mean ± SEM. The null hypothesis was rejected at the 0.05 level (**).

## 3. Results and Discussion

### 3.1. Three miR-7 Loci Are Expressed in Endocrine Cells

**Figure 1:**
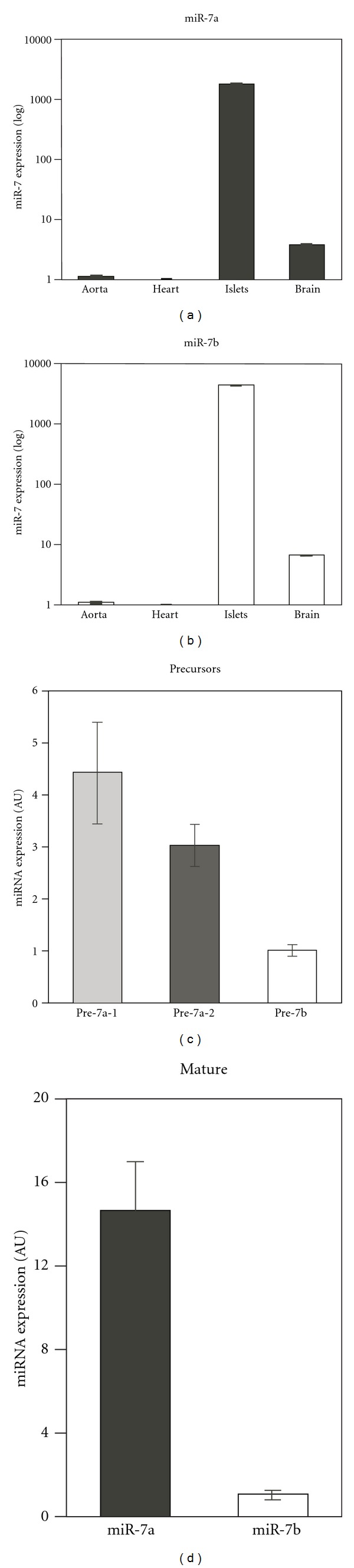
Three miR-7 genes are expressed in beta cells. (a) and (b) Taqman qPCR analysis for mature miR-7a and miR-7b in several tissues: the aorta and pulmonary artery, heart, isolated islets, and brains from three months old mice. Data normalized to sno234. (c) qPCR analysis for miR-7 precursors reveals expression in beta-TC cultured cells. Data normalized to GAPDH and 18S. *n* = 4 independent measurements, duplicates each. (d) Taqman qPCR analysis to mature miR-7a and miR-7b in beta-TC cultured cells. Data normalized to sno234. *n* = 4 independent measurements in duplicates. Error bars represent ± SEM.

Since miR-7 gene has three genomic copies in mouse and human, we first determined which of them is expressed in endocrine cells. As previously shown [19], miR-7a and miR-7b were highly and specifically expressed in islets of Langerhans, relative to other organs such as heart and brain (Figures 1(a) and 1(b)). The precursors transcribed from the three different loci can be distinguished by quantitative real-time PCR (qPCR). Therefore, we designed specific primers for each of miR-7 precursors (for oligo sequences, see Table  S1) and performed a qPCR study in BetaTC3 cell line. This analysis revealed that all three miR-7 genes are expressed in cultured beta cells (Figure 1(c)). Consistent with these results, qPCR for the mature miR-7a and miR-7b revealed expression of both forms of miR-7 in beta cells (Figure 1(d)). Taken together, the analysis of miR-7 expression suggests that the three miR-7 loci are activated in beta cells and are responsible for the overall high expression of the mature miR-7. 

### 3.2. Sequence Analysis of Potential miR-7 Regulatory Regions

Comparative genomics is commonly used to identify conserved sequences of functional importance [12, 20]. Therefore, we compared the region upstream of the pre-miR-7 sequences among several vertebrate orthologs and identified three highly conserved sequences, which may be potentially functional promoter regions. Trimethylation of lysine 4 of histone 3 (H3K4) is involved in activation of transcription and marks the position of transcriptional start site of many genes, including insulin [21]. Accordingly, we identified typical H3K4 methylation pattern [22, 23], indicating possible transcriptional start sites, at the vicinity of miR-7 genomic regions.

**Figure 2:**
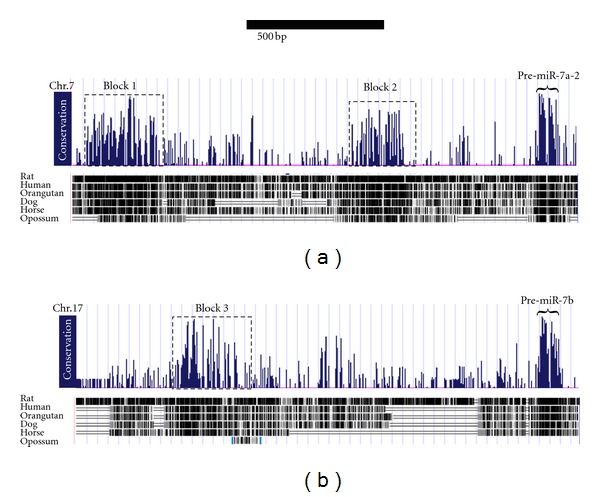
Conserved sequences upstream of miR-7 genes. Screen capture from the UCSC Genome Browser reveals fragments of Mus musculus chromosome 7 (a) and chromosome 17 (b). Tracks for conservation within mammals and the annotation of the pre-miR-7 sequences are depicted. Dashed squares demarcate the sequences that were further investigated. Scale bars represent 500 bp.

For miR-7a-2 we identified two conserved sequences: the 256 bp long “block 1” and 206 bp “block 2,” which are located 1420 bp and 450 bp upstream of the miR-7a-2 hairpin, respectively (Figure 2(a)). For miR-7b, a single 156 bp long “block 3” conserved sequence was identified, positioned 1,235 bp upstream of the miR-7b hairpin (Figure 2(b)). miR-7a-1 was omitted from this analysis since it is embedded in an intron of the Hnrnpk gene and is likely regulated by the promoter of the host gene.

### 3.3. Activity of the miR-7 Promoters in Cultured Cells

To functionally characterize miR-7 promoters, the fragments consisting of blocks 1–3 were fused separately to a firefly luciferase reporter gene, on a promoterless plasmid (pGL3-basic), and transfected into the pancreatic beta cell line MIN6 and the embryonic kidney line HEK 293. 

**Figure 3:**
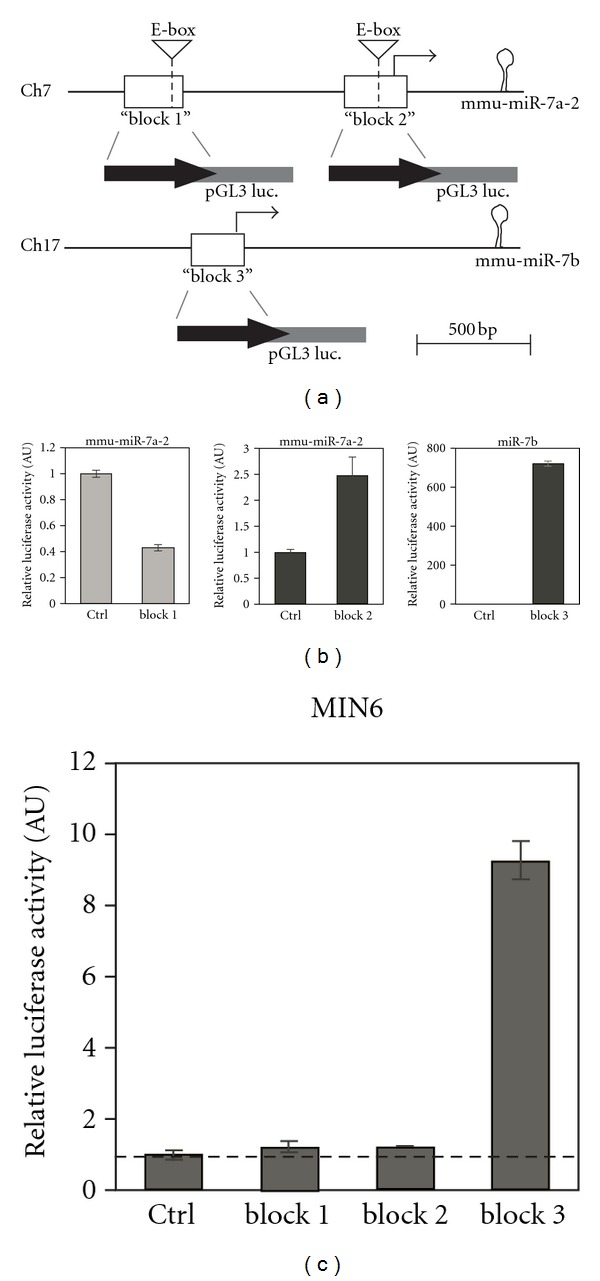
miR-7 putative promoters induce reporter activation. (a) Schematic representation of conserved sequences, upstream of pre-miR-7a-2 hairpins (“block 1” and “block 2”) and pre-miR-7b (“block 3”) that were subcloned upstream of pGL3 basic luciferase reporter. (b) Relative activation of luciferase firefly to renilla in HEK-239 cells, transfected with various reporters. pGL3-basic serves as control (“Ctrl”). (c) Relative luciferase activation in MIN6 beta cells transfected with the indicated reporters. *n* = 3 experiments, in triplicates. Error bars represent ± SEM.

In HEK-293T cells, “block 2” produced a twofold increase in luciferase activity, relative to pGL3-basic reporter (“Ctrl”), indicating weak promoter activity (Figure 3(b), middle panel). “Block 3”, on the other hand, produced a large (200-fold) increase in luciferase activity, indicating the presence of a strong promoter in this region (Figure 3(b), right panel). Surprisingly “block 1” resulted in repression of the luciferase activation, suggesting that this conserved sequence may be involved in repression of gene transcription (Figure 3(b), left panel). We then examined whether the putative promoter sequences could drive luciferase activation in MIN6 beta cells. We observed that only the DNA sequences dubbed “block 3” showed significant promoter activity (Figure 3(c)). Taken together, these results show that “block 2” and “block 3” possess weak and strong promoter activity, respectively. The activity of these fragments is not restricted to pancreatic cells, and presumably these promoter fragments require additional elements to confer selectivity in vivo. 

### 3.4. bHLH Transcription Factors Directly Induce miR-7 Promoter Activity

It has been previously shown that the bHLH transcription factors, Ngn3 and NeuroD1, play a central role in pancreas endocrine development and in mature beta-cell function, respectively. These proteins function through E-boxes (consensus sequence CAxxTG) located in target gene promoters [24]. Since two conserved E-box elements were identified in miR-7 promoter sequences (Figure 3(a)), we tested whether Ngn3 and NeuroD1 expression regulates miR-7 promoter activity.

**Figure 4:**
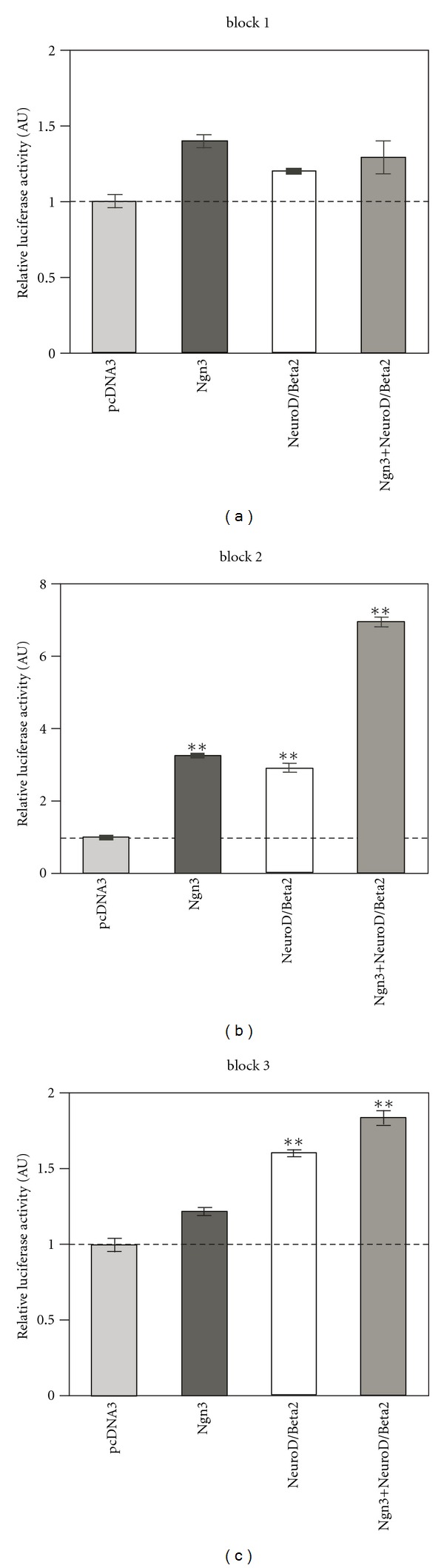
Transactivation of miR-7 reporters in HEK 293T. (a) The luciferase activity of a reporter driven by “block 1” did not demonstrate response to Ngn3 or NeuroD/Beta2. However, reporters driven by “block 2” are induced by both Ngn3 and NeuroD/Beta2 (b). Highest luciferase response is induced by a combination of Ngn3 and NeuroD/Beta2. (c) “Block 3” transcription is transactivated only by NeuroD/Beta2 but not by Ngn3. Luciferase activity normalized to A20-Renilla expression and to transactivation by a control pcDNA3-empty vector. *n* = 3 experiments, in triplicates. Error bars represent ±SEM (**P < 0.05).

For this, we performed transactivation experiments, in which luciferase reporter constructs were cotransfected into HEK-293T cells in the presence of expression vectors encoding the endocrine transcription factors. These analyses showed activation of “block 2” promoter in response to both NeuroD/Beta2a and Ngn3. In the presence of both factors, an additive effect was observed (Figure 4(b)). With “block 3”, NeuroD/Beta2 produced significant activation, whereas Ngn3 had little or no effect. The expression of block 1-containing promoter was not significantly affected by any of the co-transfected transacting factors.

Our study, therefore, suggests that the sequence upstream of miR-7b is able to activate transcription in cultured beta cells and can be activated by the endocrine transcription factor NeuroD/Beta2. The block 2 region upstream of miR-7a-2 can be activated both by NeuroD/Beta 2 and by Ngn3. However, this sequence shows very low promoter activity in the beta cell line MIN6 and therefore probably works in concert with other elements in order to transcribe the miR-7a-2 locus in beta cells.

A single ancestral miR-7 gene in invertebrates has undergone genomic duplication in the vertebrate clade. While duplication of genes often leads to functional divergence of each locus, our data suggest that miR-7 expression from three independent loci contributes primarily to higher levels of expression. Furthermore, coregulation of miR-7a-2 and miR-7b by NeuroD/Beta2 suggests that these genes respond to similar transacting factors in the endocrine pancreas.

### 3.5. Endogenous miR-7 Expression Is Activated by NeuroD/Beta2 in Cultured Beta Cells

**Figure 5:**
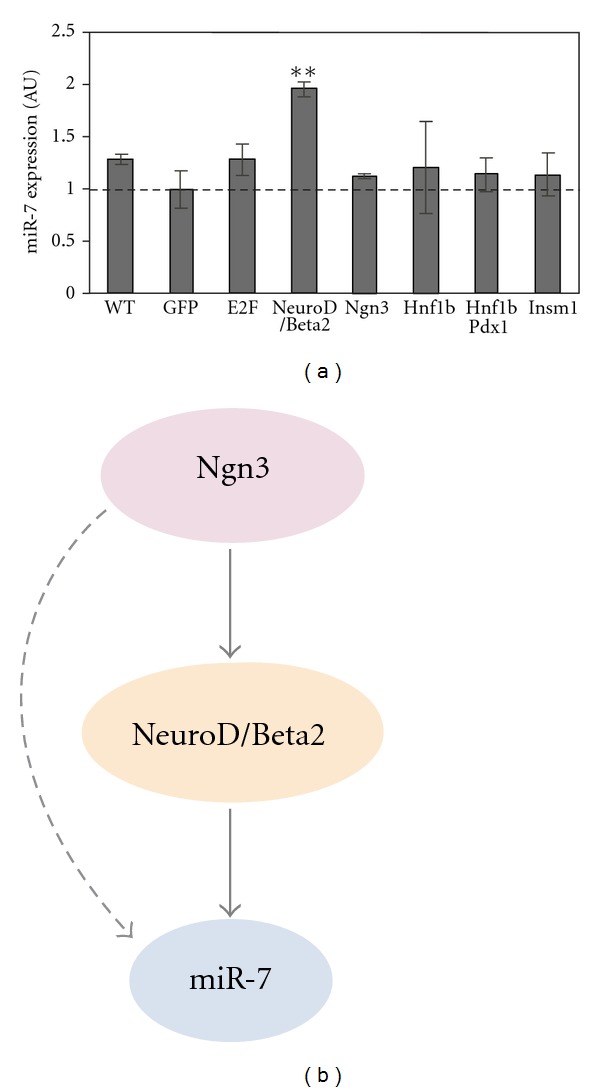
NeuroD/Beta2 regulates miR-7. (a) miR-7 expression in MIN6 cells (passages 22–24), transfected with expression vectors for various transcription factors. Endogenous miR-7 expression is upregulated upon NeuroD/Beta2 introduction, relative to GFP-expressing control vector. (b) A Schema describing miR-7 regulators.

Finally, we determined whether expression of endogenous miR-7 can be modified by introducing endocrine transcription factors into beta cell lines. To this end, we transfected MIN6 cells with expression vectors for Pdx1, Ngn3, NeuroD/Beta2, Hnf1b, Insm1, and E2A. Of these, miR-7 expression was induced in cultured beta cells only by NeuroD/Beta2 (Figure 5(a)). This is consistent with the results obtained from reporter experiments and suggests that selective expression of miR-7 is controlled by lineage-specific transcription factors, primarily, NeuroD/Beta2. This supports the hypothesis that miR-7 itself may be a component of this cascade with functional roles in controlling endocrine cell development at a posttranscriptional level (see model in Figure 5(b)).

## 4. Conclusions

During development, Ngn3 induces endocrine cell differentiation by upregulating transcription factors such as NeuroD/Beta2, Pax4, Arx, and Pax6. Indeed, loss of Ngn3 causes blockade in endocrine differentiation [5]. At the same time, Ngn3 also upregulates miR-7 expression through its effector, the transcription factor NeuroD/Beta2, that is likely involved in maintaining miR-7 expression also in mature cells.

In summary, our analysis identifies miR-7 as a novel component downstream of Ngn3 and NeuroD, embedded within the transcription factor network regulating pancreas development. Further dissection of the transcriptional mechanisms controlling expression of miR-7 and other endocrine miRNAs will contribute substantially to our overall understanding of the role of miRNA in pancreas development and function.

## Supplementary Material

Supplementary Material: Primers used in this study.

## References

[1] Lyttle BM, Li J, Krishnamurthy M (2008). Transcription factor expression in the developing human fetal endocrine pancreas. *Diabetologia*.

[2] Martin M, Hauer V, Messmer M, Orvain C, Gradwohl G (2007). Transcription factors in pancreatic development: animal models. *Endocrine Development*.

[3] Bonal C, Herrera PL (2008). Genes controlling pancreas ontogeny. *International Journal of Developmental Biology*.

[4] Gu G, Dubauskaite J, Melton DA (2002). Direct evidence for the pancreatic lineage: NGN3+ cells are islet progenitors and are distinct from duct progenitors. *Development*.

[5] Gradwohl G, Dierich A, LeMeur M, Guillemot F, Guillemot F (2000). Neurogenin3 is required for the development of the four endocrine cell lineages of the pancreas. *Proceedings of the National Academy of Sciences of the United States of America*.

[6] Ackermann AM, Gannon M (2007). Molecular regulation of pancreatic *β*-cell mass development, maintenance, and expansion. *Journal of Molecular Endocrinology*.

[7] Bartel DP (2004). MicroRNAs: genomics, biogenesis, mechanism, and function. *Cell*.

[8] Hornstein E, Shomron N (2006). Canalization of development by microRNAs. *Nature Genetics*.

[9] Stark A, Brennecke J, Bushati N, Russell RB, Cohen SM (2005). Animal microRNAs confer robustness to gene expression and have a significant impact on 3′UTR evolution. *Cell*.

[10] Li X, Cassidy JJ, Reinke CA, Fischboeck S, Carthew RW (2009). A microRNA imparts robustness against environmental fluctuation during development. *Cell*.

[11] Saini HK, Griffiths-Jones S, Enright AJ (2007). Genomic analysis of human microRNA transcripts. *Proceedings of the National Academy of Sciences of the United States of America*.

[12] Avnit-Sagi T, Kantorovich L, Kredo-Russo S, Hornstein E, Walker MD (2009). The promoter of the pri-miR-375 gene directs expression selectively to the endocrine pancreas. *PLoS One*.

[13] Wienholds E, Kloosterman WP, Miska E (2005). Cell biology: microRNA expression in zebrafish embryonic development. *Science*.

[14] Landgraf P, Rusu M, Sheridan R (2007). A mammalian microRNA expression atlas based on small RNA library sequencing. *Cell*.

[15] Correa-Medina M, Bravo-Egana V, Rosero S (2009). MicroRNA miR-7 is preferentially expressed in endocrine cells of the developing and adult human pancreas. *Gene Expression Patterns*.

[16] Bravo-Egana V, Rosero S, Molano RD (2008). Quantitative differential expression analysis reveals miR-7 as major islet microRNA. *Biochemical and Biophysical Research Communications*.

[17] Lewis BP, Shih IH, Jones-Rhoades MW, Bartel DP, Burge CB (2003). Prediction of microRNA targets. *Cell*.

[18] Kefas B, Godlewski J, Comeau L (2008). microRNA-7 inhibits the epidermal growth factor receptor and the akt pathway and is down-regulated in glioblastoma. *Cancer Research*.

[19] Joglekar MV, Joglekar VM, Hardikar AA (2009). Expression of islet-specific microRNAs during human pancreatic development. *Gene Expression Patterns*.

[20] Pennacchio LA, Rubin EM (2001). Genomic strategies to identify mammalian regulatory sequences. *Nature Reviews Genetics*.

[21] Francis J, Chakrabarti SK, Garmey JC, Mirmira RG (2005). Pdx-1 links histone H3-Lys-4 methylation to RNA polymerase II elongation during activation of insulin transcription. *The Journal of Biological Chemistry*.

[22] Harrow J, Denoeud F, Frankish A (2006). GENCODE: producing a reference annotation for ENCODE. *Genome Biology*.

[23] Flicek P, Amode MR, Barrell D (2011). Ensembl 2011. *Nucleic Acids Research*.

[24] Glick E, Leshkowitz D, Walker MD (2000). Transcription factor BETA2 acts cooperatively with E2A and PDX1 to activate the insulin gene promoter. *The Journal of Biological Chemistry*.

