# Supplementary material for: Regulation of Pancreatic microRNA-7 Expression
Source: Exp Diabetes Res. 2012 May 17;2012:695214. doi: 10.1155/2012/695214 (PMC3362837; doi:10.1155/2012/695214)
Supplement: Supplementary file 1 — Supplementary Material: Primers used in this study. [file 695214.f1.pdf]

Table S1. Primers used in this study

Gene name	Forward Primer	Reverse Primer
Pre-7b	ACGTGAGCCAGTGCTATGTG	TAGGAGTCCACGCTATGAGG
Pre-7a-1	CTGTAGAGGTGGCCTGTGC	TGGCCTAGTTCTGTGTGGAA
Pre-7a-2	AGCCCCGTTTGGAAGACTA	TGAAATGACCAGCACCATGT
Block1 promoter fragment	ATATTCTCCAGCCTGCCTCA	GAAAAGTGACCTGCCCTTGA
Block2 promoter fragment	GGAGGGGTAGAGCAAAGAGG	TCTGGGCCAGCCTCTAATTG
Block3 promoter fragment	TTTTGGACGGATGCCTGACT	CTTTATGCGCTTAAGGGGAAG
